# Greek Yogurt Compared with Whey Protein Supplementation in Adolescent Athletes Throughout a Competitive Season

**DOI:** 10.1016/j.tjnut.2026.101524

**Published:** 2026-04-05

**Authors:** Madison Bell, Pedro Henrique Narciso, Ethan Miskolczi, Maria I Retsidou, Brian D Roy, Andrea R Josse, Bareket Falk, Panagiota Klentrou

**Affiliations:** 1 Department of Kinesiology, Brock University, St. Catharines, ON, Canada; 2 Centre for Bone and Muscle, Brock University, St. Catharines, ON, Canada; 3 Faculty of Science, Thompson Rivers University, Kamloops, BC, Canada; 4 School of Kinesiology and Health Science, York University, ON, Canada

**Keywords:** dairy, bone formation, bone resorption, ILs, TNF-α

## Abstract

**Background:**

Protein intake during adolescence may influence bone development and immune status, yet the comparative effects of wholefood protein sources compared with protein isolates in adolescent athletes remain unclear.

**Objectives:**

To compare the effects of Greek yogurt (GY) and whey protein (WP) supplementation on bone and inflammation markers in adolescent athletes throughout a competitive season.

**Methods:**

Athletes completed an initial control period on their habitual diets (weeks 0‒8), followed by randomization to GY (*n* = 24; 15.8 ± 1.1 y; 11 females) or WP (*n* = 23; 16.0 ± 1.4 y; 10 females) for a 16-wk intervention (weeks 8‒24). GY consumed 2 servings per day of 175 g GY (17 g protein); WP received an isonitrogenous WP supplement. Blood samples and body composition assessments were obtained at weeks 0, 8, 16, and 24.

**Results:**

Procollagen type I N-terminal propeptide, insulin-like growth factor-1, and receptor activator of nuclear factor κB ligand remained stable. Osteocalcin and osteoprotegerin showed sex-specific between-group differences: osteocalcin declined throughout weeks 0‒24 in GY males, whereas osteoprotegerin declined during weeks 0‒16 in GY females and was elevated at the end of the control period in WP females. C-terminal telopeptide of type I collagen and sclerostin showed intervention group-dependent, but not sex-dependent, differences. C-terminal telopeptide of type I collagen increased transiently from week 8 to week 16 and returned to baseline by week 24 only in GY. Sclerostin concentrations fluctuated, and at 24 wk, were not different from baseline in GY but were higher than baseline in WP. At week 16, interleukin (IL)-1β increased in WP, and IL-6 decreased in GY. IL-10 and tumor necrosis factor α increased during the control period and decreased with WP only in females.

**Conclusions:**

Increased protein intake, independent of source, was associated with modest, often sex-specific fluctuations in bone and inflammatory markers in adolescent athletes, potentially influenced by growth and training-related factors across the competitive season.

This trial was registered at clinicaltrials.gov as NCT05922462.

## Introduction

It is well established that high-impact sports, such as running and soccer, induce persistent, multidirectional mechanical loading on the skeleton, which enhances bone turnover and bone mineral density in adolescent and adult athletes [1,2]. Adequate bone formation is particularly critical during adolescence as >90% of peak bone mass is achieved by 18 y [3], highlighting the importance of diet and mechanical loading during this time frame [4]. Despite the benefits of mechanical loading, extended periods of intense training may exacerbate inflammation, elevating the risk of bone stress injuries, stress fractures, and other chronic skeletal impairments [5,6].

Current nutritional strategies for athletic youth predominantly emphasize isolated dairy-derived whey protein (WP) supplements. WP isolate is rapidly digested and contains high concentrations of branched-chain amino acids [7,8]. WP isolate supplementation has been associated with increased markers of bone formation, such as osteocalcin (OC) and insulin-like growth factor 1 (IGF-1), an anabolic hormone involved in musculoskeletal growth and bone development, and may influence bone remodeling via the osteoprotegerin (OPG)/receptor activator of nuclear factor κΒ ligand (RANKL) signaling pathway [9]. Among adolescent female athletes, WP supplementation has been shown to elevate the anti-inflammatory cytokine IL-10 in response to intensified training but had no short-term effect on IL-6 and pro-inflammatory cytokines, including IL-1β and TNF-α [10,11]. However, because WP lacks the diverse micronutrient and bioactive profile found in wholefood dairy products like Greek yogurt (GY), its anti-inflammatory potential may be comparatively limited in adolescent populations [10].

Dairy products are well-suited to address the need for optimizing nutritional strategies. Beyond being rich sources of calcium and high-quality protein, dairy foods also contain bioactive peptides, lactoferrin, and precursors to glutathione, all of which may contribute to anti-inflammatory and antioxidant effects [[12], [13], [14]]. Fermentation further enhances the nutritional value of dairy by improving nutrient absorption in the gut and modulating immune responses through bacterial cultures such as Lactobacillus, which help maintain gut integrity and influence cytokine signaling [15]. GY, in particular, is a casein-rich, fermented dairy product that has been shown to improve bone formation and modulate inflammation when consumed in conjunction with exercise training in both young adult nonathletic males [16] and adolescent female athletes [17]. Similarly, acute milk consumption, a dairy source composed of both whey and casein proteins, has been found to reduce post exercise inflammation in young females by lowering IL-1β and IL-6 [18].

Collectively, these immunomodulatory properties suggest that wholefood dairy products like GY may offer benefits that extend beyond conventional macronutrient supplementation, particularly in contexts such as exercise recovery, inflammation control, and bone health. However, there is a notable lack of studies directly comparing the effects of long-term supplementation of wholefood dairy products, such as GY, compared with isolated dairy-derived protein supplements, such as WP, on markers and regulators (i.e., osteokines) of bone turnover and inflammation in adolescent athletes. Additionally, sex-specific responses remain underexplored, despite evidence that female athletes may be particularly vulnerable to inflammation-related bone imbalances [19]. Therefore, this study examined the effects of GY compared with isonitrogenous WP supplementation on circulating bone turnover markers, osteokines, and inflammatory cytokines in female and male adolescent athletes throughout their competitive season. The selected markers of bone turnover reflect the dynamic processes of bone formation and resorption. OC, a general bone turnover marker, and procollagen type I N-terminal propeptide (P1NP), a byproduct of collagen synthesis, indicate osteoblast activity, whereas C-terminal telopeptides of type I collagen (CTX-I), a byproduct of collagen breakdown, reflect osteoclast activity [[19], [20], [21]]. In terms of osteokines, sclerostin (SOST) inhibits bone formation, RANKL promotes osteoclastogenesis, and OPG acts as a downregulator of bone resorption by inhibiting RANKL. Although higher formation markers and lower resorption markers are generally interpreted as favorable for bone health, interpretation during adolescence is more complex as bone turnover is naturally elevated during periods of rapid skeletal growth [3,21,22]. Nevertheless, we hypothesized that GY would result in higher resting concentrations of bone formation markers and lower resting concentrations of bone resorption markers compared with WP in adolescent athletes of both sexes. We also hypothesized that GY would exert a stronger anti-inflammatory effect than WP in adolescent athletes of both sexes.

## Methods

The data presented herein are part of a broader randomized controlled clinical trial approved by the Research Ethics Board at Brock University in Ontario, Canada (research ethics board number: 22-303). The trial adhered to the ethical guidelines outlined in the latest Tri-Council Policy Statement: Ethical Conduct for Research Involving Humans (2nd edition) governing research involving human participants in Canada. The complete trial is registered on clinicaltrials.gov (identifier: NCT05922462). This study presents data from the adolescent athlete cohort that were collected during phase 2 of the trial. The results from the adult athlete cohort collected during phase 1 of the trial are published by Bell et al. [20].

### Participants

Prior to study commencement, a priori sample size calculation was performed for repeated measures analysis of variance (within-between interaction) in G∗Power using the mean effect size from 2 previous studies on the effects of GY supplementation on bone markers and cytokines in youth athletes [17,21]. This calculation determined that a total sample size of *n* = 24 would be required to detect an average effect size of 0.25 with a power 1-β = 0.80, and a probability level *α* = 0.05. To account for attrition during the 24 wk of the study, a total of 50 14‒17-y-old adolescent athletes, split between males (*n* = 26) and females (*n* = 24), were initially recruited to participate in the study. For consistency, athletes were recruited from a range of high-impact competitive sports based in the Niagara region of Ontario, including soccer, volleyball, basketball, track-and-field, and lacrosse. To be eligible, participants had to be actively engaged in training and competition throughout the study period. Individuals were excluded if they had injuries that interfered with training, known dairy allergies, or a diagnosis of lactose intolerance. Informed written assent and parental consent were obtained from all participants and their parents or guardians prior to study enrollment.

Three participants (all females) withdrew from the study after the week 8 measurements and around week 9 (i.e., during the first week of intervention) as they could not consistently maintain the supplementation requirements. Those participants’ data were removed from the analysis, resulting in a final sample of 47 athletes evenly randomly assigned into the 2 intervention arms (see details under experimental design). Specifically, 24 athletes assigned to the GY group (13 males; 11 females), and 23 athletes assigned to the WP group (13 males; 10 females) completed the study.

### Experimental design

The randomization protocol, intervention schedule, and experimental procedures were previously described in Bell et al. [20]. For the adolescent cohort, the randomized controlled trial spanned 24 wk during the athletes’ active competitive season; April 2024 to September 2024 for outdoor sports (soccer, track-and-field, and lacrosse) and November 2024 to April 2025 for indoor sports (basketball and volleyball). Based on the primary sport reported by participants, 37 athletes were classified as primarily indoor sport athletes and 10 as primarily outdoor sport athletes, although most sports involved elements of both indoor and outdoor training. Using a parallel design, the study protocol consisted of an initial 8-wk control period, spanning from week 0, i.e., the beginning of the competitive season, to week 8, followed by a 16-wk intervention period, covering the period from week 8 to week 24, i.e., near the end of the competitive season. During the initial 8-wk control condition, all participants followed their habitual diets. Subsequently, participants were randomly assigned (1:1 allocation ratio) using a computer-generated randomization sequence to 1 of 2 trial arms supplementing their habitual diets with 2 daily servings of either GY or of isonitrogenous/isoenergetic WP for the 16-wk intervention period. Randomization was also stratified by sex to ensure a similar number of male and female participants in each group.

The study was conducted as a single-blind trial, in which investigators remained unaware of group assignments throughout the intervention. To maintain blinding, supplements were distributed weekly by independent research assistants who were not involved in data collection or analysis. Although participants were aware of the type of supplement they were consuming (i.e., GY compared with WP), those in the WP group were blinded to the specific brand and nutrient composition of the WP isolate used, as this information was not disclosed to them.

### Nutritional intervention

To maintain ecological validity, the 16-wk intervention phase (weeks 8–24) was designed to reflect typical dietary habits by adding, rather than replacing, foods in the regular diets of participants. Athletes were instructed to consume 2 daily servings of either GY or WP alongside their usual meals. Those in the GY group received 2 175 g servings per day of Oikos high protein, flavored GY (Danone), providing 17 g of protein, 0% fat, ∼130 kcal, and 225 mg of calcium per serving. Participants assigned to the WP group consumed 2 servings per day of ISO Advanced WP isolate powder (ProLine) dissolved in water. Each serving provided 17 g of protein, 0% fat, ∼70 kcal, and 76 mg of calcium. The WP supplement was composed of 100% crossflow micro-filtered WP isolate (90% purity). To enhance the feasibility and real-world relevance of the protocol, participants were encouraged, but not required, to space their servings between morning and evening. Timing was flexible to accommodate training and competition schedules. Additionally, athletes could choose from a selection of flavors (GY: vanilla or berry; WP: vanilla, chocolate, or strawberry-banana), with all options standardized for nutrient content.

### Measurements

Measurements of height, body mass, and body composition were collected at all 4 study time points (weeks 0, 8, 16, and 24). Standing height was assessed using a portable stadiometer (Seca 213, CME Corp.) and recorded to the nearest 0.1 cm. Body mass and composition were measured via bioelectrical impedance analysis using the InBody system (BioSpace), which provided estimates of total body mass, as well as fat mass and lean mass used to calculate percent body fat (%BF). To ensure consistency in bioelectrical impedance analysis measurements, participants were asked to drink 1 cup of water prior to arriving at the laboratory and to empty their bladder immediately before assessment.

Training volume and patterns were documented through training logs collected at each laboratory visit, providing a comprehensive overview of participants’ training regimens. Dietary intake and energy balance were assessed using the automated self-administered 24-h dietary recall (ASA24) at all 4 laboratory visits. The ASA24 provided detailed, standardized dietary intake data for each time point, capturing macronutrient intake, calcium consumption, and supplement use throughout the study period [22]. Participants completed the ASA24 recalls with guidance to ensure accuracy and consistency. Finally, compliance with supplementation was evaluated through self-report at each laboratory visit during the intervention (weeks 16 and 24) using a 10-point scale, where a score of 10 indicated perfect adherence, and 0 indicated no adherence.

### Blood analysis

To assess the impact of the intervention compared with the control period on bone turnover and inflammation, resting blood samples were collected in the morning between 08:00 and 10:00 at 4 time points: pre-control (week 0, i.e., beginning of competitive season), post control/pre-intervention (week 8), mid-intervention (week 16), and post intervention (week 24, i.e., end of season). Samples (10 mL of blood) were collected by a certified phlebotomist from the antecubital vein after an overnight fast of 10–12 h and ≥24 h after the last training session or game. Blood was drawn into BD Vacutainer gold-top tubes (with clot activator and gel for serum separation), and BD Vacutainer purple-top tubes (containing EDTA as an anticoagulant). Blood samples sat for 30 min at room temperature before being centrifuged at 3000 × *g*, 4°C, 10 min in a benchtop centrifuge (Allegra ZIR centrifuge, Beckman Coulter). The resulting serum and plasma were then aliquoted into air-tight cryotubes (Axygen MCT-150-C, Corning Inc.) and stored at −80°C until analysis.

The circulating concentrations of markers of bone turnover were analyzed by Eve Technologies Corporation. P1NP and CTX-I analyses were performed on the PerkinElmer EnSpire 2300 Multilabel Reader System. P1NP was measured using the Human P1NP ELISA Kit (Elabscience Biotechnology Inc.) with assay sensitivity of 9.38 pg/mL and an intra- and interassay coefficients of variation (%CV) <10 and <15, respectively. CTX-I was measured using the Human CTX-I ELISA Kit (Elabscience Biotechnology Inc.) with assay sensitivity of 0.09 ng/mL and an intra- and interassay %CV <10 and <15, respectively.

OC, OPG, SOST, IL-1β, and IGF-1 were measured using the Luminex 200 system (Luminex Corporation) with Bio-Plex Manager software (version 6.1, Bio-Rad Laboratories Inc.). This system allowed the simultaneous measurement of OC, OPG, SOST, and IL-1β using a Human Bone Custom Assay (MilliporeSigma). Assay sensitivities of these markers ranged from 1.9 pg/mL to 68.5 pg/mL, the intra-assay %CV was <10, and the interassay %CV was <15. IGF-1 was measured using a Human IGF Custom Assay (MilliporeSigma) with a sensitivity of 15 pg/mL for IGF-1 and the intra- and interassay %CV <10 and <15, respectively.

RANKL analysis was performed using the MESO QuickPlex SQ 120MM instrument with Methodical Mind and DISCOVERY Workbench software (version 4.0, Meso Scale Discovery). RANKL concentrations were measured using the Human MSD RANKL Custom Assay (Human RANKL/TNFSF11, K151M7K-1, Meso Scale Discovery). The average assay sensitivity for this biomarker is 1.8 pg/mL, and an intra- and interassay %CV <10 and <15, respectively. All samples passed internal quality checks, and duplicates met consistency thresholds.

The circulating concentrations of TNF-α, IL-6, and IL-10 were analyzed on an ELLA automated Enzyme-Linked Immunosorbent Assay (ELISA) platform (Bio-Techne) using a commercially available Simple Plex Human Multianalyte Panel (catalog number SPCKE-PS-010895; ProteinSimple). All samples of the same participant were analyzed on a single plate. The reported assay sensitivity for this panel is 0.30 pg/mL for TNF-α, 0.11 pg/mL for IL-6, and 0.17 pg/mL for IL-10. The intra-assay %CV measured in-house were 2.54%, 4.60%, and 6.69% for TNF-α, IL-6, and IL-10, respectively.

### Statistical Analysis

Data were first assessed for normality using a combination of visual methods (histograms, Quantile-Quantile plots), skewness and kurtosis values, and standardized *z*-scores (threshold: ±3). Of the 188 expected blood samples (47 participants × 4 blood samples), 23 were missed, resulting in a final dataset of 165 samples for blood-based analyses. In addition, a total of 33 outlier values were excluded from the analysis of P1NP (*n* = 9/165), SOST (*n* = 2/165), IL-1β (*n* = 11/165), IL-10 (*n* = 10/165), and TNF-α (*n* = 1/165) based on IQR. These outliers were defined as data points that fell below the lower fence (Q1 ‒ 1.5 × IQR**)** or above the upper fence (Q3 + 1.5 × IQR). As such, the number of observations slightly varied across specific biomarkers.

Linear mixed-effects models were employed to test the main effects of time (repeated measure), group (GY compared with WP), and sex (male compared with female), as well as their interactions, on training volume, dietary intake, body composition, and biomarkers. For the models of body composition, bone markers, and inflammatory cytokines, we included training volume as a covariate and participant identification as a random effect to account for within-subject variability. The baseline values of each marker were also included as a covariate in the biomarker models. When significant time effects or interactions were identified, post hoc pairwise comparisons were conducted with false discovery rate correction to control for multiple testing. All analyses were performed using lme4 package in R (Bell Laboratories, Lucent Technologies), with statistical significance set at *P* < 0.05. Data are presented as mean ± SD.

## Results

### Nutritional and body composition variables

**FIGURE 1:**
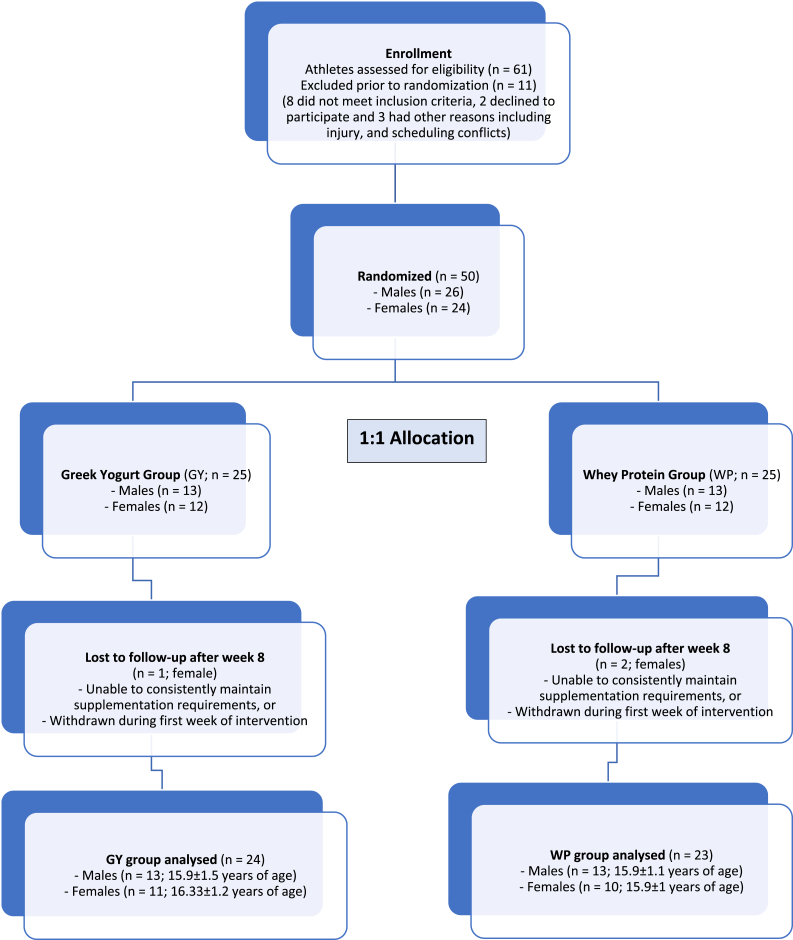
Participant flow (recruitment, randomization, completers and analysis) through the study.

Figure 1 shows the participant flow (recruitment, randomization, completers, and analysis) through the study. A total of 47 athletes completed the study; 24 were in the GY group, 13 males, 15.9 ± 1.5 y of age, and 11 females, 16.3 ± 1.2 y of age, and 23 were in the WP group (13 males, 15.9 ± 1.1 y of age; 10 females, 15.9 ± 1 y of age). Compliance with supplementation remained consistent throughout the intervention period, with no meaningful changes observed over time in any group. Mean ± SD compliance scores out of 10 for male athletes in the GY group were 7.8 ± 1.4 at week 16 and 7.6 ± 1.8 at week 24 (Fisher-statistic [*F*] = 0.64, *P* = 0.447). Compliance scores for female athletes in the GY group were identical at week 16 and week 24 (8.7 ± 1.5; *F* = 0.00, *P* = 1.00). In the WP group, male athletes reported compliance scores of 7.1 ± 1.8 at week 16 and 7.5 ± 1.4 at week 24 (*F* = 0.48, *P* = 0.504), whereas female athletes reported scores of 7.1 ± 1.5 at week 16 and 7.2 ± 1.5 at week 24 (*F* = 1.00, *P* = 0.351).

Participants’ dietary intakes were recorded at weeks 0, 8, 16, and 24 and are presented in Table 1. We observed a time × group × sex interaction in dietary energy intake (*F* = 2.71, *P* = 0.048). Energy intake decreased from baseline to the end of the intervention (weeks 0‒24, *P* = 0.009) in GY males, whereas it declined mid-season (weeks 8 and 16, *P* = 0.029 and *P* = 0.001, respectively) and rebounded by week 24 (*P* = 0.002) in WP males. No such differences across time were seen in females (Table 1). A time × group × sex interaction was also found in carbohydrate intake (*F* = 3.43, *P* = 0.019), which was higher at week 0 compared with all other times in GY males and higher than week 8 and week 24 in WP females (Table 1). Protein intake showed a significant sex main effect (*F* = 4.38, *P* = 0.040), reflecting higher intake in males compared with females, with no significant changes over time (Table 1). We observed a significant time × group interaction for fat intake (*F* = 2.93, *P* = 0.036), reflecting lower fat intake at week 24 only within the GY group with males and females combined (Table 1). There was a significant time × group × sex interaction in vitamin D intake (*F* = 11.47, *P* = 0.001), reflecting a drop at week 8 in GY males and at week 16 in WP males (Table 1). Calcium intake was higher in males compared with females (sex main effect, *F* = 5.95, *P* = 0.019), with no significant changes across time and no interactions (Table 1).

**TABLE 1:** Daily energy, carbohydrate, fat, protein, vitamin D and calcium intake at weeks 0, 8, 16, and 24 for male and female athletes in the Greek yogurt and whey protein groups

		Week 0	Week 8	Week 16	Week 24
Energy intake (kcal)	GY	M: 2788 ± 540 F: 1732 ± 636	M: 2452 ± 1020 F: 2001 ± 515	M: 2456 ± 593 F: 2097 ± 856	M: 2089 ± 916^2^ F: 1863 ± 705
	WP	M: 2930 ± 663 F: 2351 ± 186	M: 2394 ± 699^2^ F: 1721 ± 190	M: 2116 ± 830^2^ F: 2120 ± 537	M: 2881 ± 669^3^^,^^4^ F: 1990 ± 134
Carbohydrate intake (g/kg)	GY	M: 5.2 ± 1.5 F: 3.6 ± 1.3	M: 3.8 ± 1.2^2^ F: 4.4 ± 1.4	M: 4.0 ± 1.9^2^ F: 4.1 ± 2.5	M: 3.6 ± 1.8^2^ F: 3.5 ± 1.5
	WP	M: 4.9 ± 1.1 F: 5.6 ± 0.8	M: 4.3 ± 1.6 F: 3.3 ± 0.5^2^	M: 3.9 ± 2.7 F: 4.4 ± 1.2	M: 4.1 ± 1.3 F: 3.6 ± 0.6^2^
Protein intake (g/kg)^1^	GY	M: 1.7 ± 0.6 F: 1.1 ± 0.5	M: 1.6 ± 0.8 F: 1.3 ± 0.5	M: 1.6 ± 0.6 F: 1.5 ± 0.7	M: 1.6 ± 0.4 F: 1.7 ± 0.9
	WP	M: 1.9 ± 0.7 F: 1.6 ± 0.3	M: 1.7 ± 0.7 F: 1.2 ± 0.2	M: 1.6 ± 0.8 F: 1.4 ± 0.2	M: 1.9 ± 0.8 F: 1.5 ± 0.2
Fat intake (g)	GY	M: 1.5 ± 0.7 F: 1.0 ± 0.5	M: 1.3 ± 0.7 F: 0.9 ± 0.3	M: 1.1 ± 0.4 F: 1.3 ± 0.6	M: 1.0 ± 0.5 F: 1.0 ± 0.5
	WP	M: 1.6 ± 0.7 F: 1.3 ± 0.3	M: 1.3 ± 0.5 F: 1.3 ± 0.2	M: 1.3 ± 0.8 F: 1.3 ± 0.3	M: 1.7 ± 0.6 F: 1.5 ± 0.2
Vitamin D intake (mcg)	GY	M: 9.4 ± 5.1 F: 4.6 ± 3.5	M: 5.5 ± 3.5 F: 5.1 ± 2.6	M: 8.3 ± 7.7 F: 4.8 ± 2.4	M: 6.9 ± 5.3 F: 7.1 ± 5.4
	WP	M: 8.4 ± 4.7 F: 5.7 ± 2.3	M: 7.8 ± 6.4 F: 2.7 ± 0.9	M: 3.7 ± 2.2^2^^,^^3^^,^^5^ F: 4.2 ± 2.9	M: 8.0 ± 6.2 F: 2.3 ± 0.9
Calcium intake (mg)^1^	GY	M: 1328 ± 331 F: 731 ± 459	M: 1091 ± 660 F: 993 ± 271	M: 1336 ± 725 F: 890 ± 458	M: 983 ± 662 F: 864 ± 426
	WP	M: 1171 ± 652 F: 986 ± 240	M: 1018 ± 405 F: 893 ± 151	M: 1064 ± 733 F: 982 ± 385	M: 1541 ± 880 F: 909 ± 373

Data are displayed as mean ± SD. GY group (*n* = 24, 13 males); WP group (*n* = 23, 13 males).

Abbreviations: F, female; GY, Greek yogurt; M, male; WP, whey protein.

1 Significant higher in males across time (main sex effect).

2 Significantly lower from week 0 within sex and group (time × group × sex interaction).

3 Significantly higher from week 8 within sex and group (time × group × sex interaction).

4 Significantly higher from week 16 within sex and group (time × group × sex interaction)

5 Significantly lower from week 24 within sex and group (time × group × sex interaction).

**TABLE 2:** Height, body mass, body fat percentage, and training volume at weeks 0, 8, 16, and 24 for male and female athletes in the Greek yogurt and whey protein groups

		Week 0	Week 8	Week 16	Week 24
Height (cm)	GY	M: 177.3 ± 5.3 F: 164.5 ± 5.4	M: 177.2 ± 5.6 F: 164.7 ± 5.5	M: 177.8 ± 5.8 F: 164.9 ± 5.9	M: 177.6 ± 5.3 F: 164.7 ± 5.7
	WP	M: 178.7 ± 6.8 F: 165.2 ± 3.9	M: 178.3 ± 6.3 F: 165.3 ± 4.0	M: 177.9 ± 6.1 F: 165.2 ± 3.9	M: 178.4 ± 6.5 F: 165.1 ± 3.8
Body mass (kg)	GY	M: 71.2 ± 11.7 F: 67.1 ± 16.5	M: 74.6 ± 10.3^2^ F: 63.1 ± 11.5	M: 72.8 ± 9.5^2^ F: 68.0 ± 17.2	M: 74.9 ± 10.9^2^ F: 69.4 ± 17.9
	WP	M: 72.0 ± 12.7 F: 59.4 ± 9.5	M: 72.0 ± 12.3^2^ F: 61.2 ± 9.5	M: 72.7 ± 11.9^2^ F: 59.8 ± 9.7	M: 73.1 ± 13.4^2^ F: 61.1 ± 11.2
Body fat (%)^1^	GY	M: 12.2 ± 3.9 F: 26.6 ± 10.0	M: 14.5 ± 5.4 F: 25.5 ± 6.9	M: 13.2 ± 2.5 F: 26.4 ± 8.9	M: 13.5 ± 3.8 F: 26.2 ± 9.1
	WP	M: 12.3 ± 8.0 F: 22.8 ± 7.0	M: 15.2 ± 12.5 F: 24.4 ± 8.7	M: 12.3 ± 8.2 F: 21.1 ± 3.6	M: 12.0 ± 8.0 F: 18.5 ± 5.2
Training volume (h/w)∗	GY	M: 11.8 ± 5.6 F: 10.7 ± 3.2	M: 11.2 ± 5.2 F: 9.7 ± 5.0	M: 11.7 ± 4.3 F: 8.8 ± 3.7	M: 10.6 ± 5.7 F: 8.8 ± 3.4
	WP	M: 12.2 ± 5.1 F: 8.7 ± 2.2	M: 11.9 ± 6.0 F: 9.2 ± 3.8	M: 11.5 ± 4.9 F: 9.3 ± 2.6	M: 9.0 ± 4.7 F: 9.0 ± 2.5

Data are displayed as mean ± SD. GY group (*n* = 24, 13 males); WP group (*n* = 23, 13 males).

Abbreviations: F, female; GY, Greek yogurt; M, male; WP, whey protein.

1 Significantly lower in males across time (main sex effect);

2 Significant increase in males (time × sex interaction);

∗ Significant difference between week 0 and week 24 (main time effect).

Body mass had a significant time × sex interaction (*F* = 3.17, *P* = 0.027), reflecting a gradual significant increase in males, with higher body mass at weeks 8‒24 compared with week 0, whereas females had no significant change (Table 2). %BF remained stable across the intervention, with no significant main effects of time or group and no significant interactions (Table 2). However, a significant main sex effect was observed (*F* = 32.76, *P* < 0.0001), with males having consistently lower %BF than females (Table 2). Lastly, there was a significant time effect in training volume (*F* = 2.62, *P* = 0.053) that reflects an overall reduction in training volume at week 24, i.e., at the end of the intervention period, compared with baseline, i.e., the beginning of control period, for all participants, independent of supplementation group or sex (Table 2). Interestingly, although training volume was included as a covariate, it was a significant predictor only for IGF-1 concentrations (*F* = 5.68, *P* = 0.019), consistent with the load-dependent regulation of this hormone. In contrast, training volume was not significant for any of the other markers of bone turnover or inflammation.

### Markers of bone turnover

Absolute concentrations of markers of bone formation (OC, P1NP, IGF-1, and OPG) and bone resorption (CTX-I, SOST, and RANKL) across the intervention are presented in Table 3. P1NP concentrations remained stable across the intervention, with no significant main effects of time or group and no significant interactions (Table 3). However, a significant main effect of sex was observed (*F* = 7.35, *P* = 0.010), with males exhibiting consistently higher P1NP concentrations than females across all time points (Table 3). For IGF-1, no significant main effects of time, group, or sex, and no interaction effects were detected, so IGF-1 concentrations remained stable across the intervention in both groups and sexes (Table 3).

**TABLE 3:** Concentrations of markers and regulators of bone turnover at weeks 0, 8, 16, and 24 for male and female athletes in the Greek yogurt and whey protein groups

		Week 0	Week 8	Week 16	Week 24	Time	Group	Sex	Time × group	Time × group × sex
P1NP (ng/mL)	GY^1^	M: 26189 ± 13550 F: 16865 ± 5757	M: 31460 ± 15294 F: 13595 ± 7635	M: 27668 ± 12890 F: 13978 ± 4488	M: 28953 ± 14137 F: 13040 ± 2754	0.803	0.451	0.010^8^	0.127	0.315
	WP^1^	M: 21763 ± 9389 F: 11738 ± 5502	M: 23659 ± 10199 F: 9042 ± 2647	M: 25091 ± 12526 F: 11546 ± 5053	M: 21597 ± 10436 F: 13850 ± 5643					
OC (pg/mL)	GY	M: 12727 ± 6369 F: 5271 ± 1834	M: 9129 ± 5330^2^ F: 5122 ± 1594	M: 10662 ± 4989^2^^,^^3^ F: 5424 ± 1922	M: 8761 ± 3674^2^ F: 5914 ± 1585	0.010^8^	0.690	0.414	0.120	0.005^8^
	WP	M: 7996 ± 1716 F: 6240 ± 2892	M: 7833 ± 1345 F: 5544 ± 1999	M: 7481 ± 2161 F: 5585 ± 1694	M: 7749 ± 1595 F: 5811 ± 1383					
IGF-1 (pg/mL)	GY	M: 78600 ± 32094	M: 73184 ± 22598	M: 75316 ± 18121	M: 71254 ± 19777	0.121	0.936	0.276	0.543	0.234
		F: 58267 ± 18657	F: 58496 ± 19075	F: 78532 ± 25384	F: 70203 ± 20204					
	WP	M: 65636 ± 12941	M: 63240 ± 17169	M: 68288 ± 17318	M: 65662 ± 12274					
		F: 66671 ± 20551	F: 61470 ± 14061	F: 65764 ± 25781	F: 73681 ± 14833					
OPG (pg/mL)	GY	M: 303 ± 53	M: 279 ± 84	M: 283 ± 55	M: 278 ± 53	0.111	0.163	0.533	0.029^8^	0.009^8^
		F: 301 ± 37	F: 272 ± 44	F: 261 ± 35^4^	F: 291 ± 53					
	WP	M: 240 ± 37	M: 232 ± 46	M: 239 ± 66	M: 251 ± 65					
		F: 292 ± 34	F: 331 ± 59^4^	F: 284 ± 50	F: 274 ± 18					
CTX-I (pg/mL)	GY^5^	M: 0.22 ± 0.10 F: 0.22 ± 0.11	M: 0.21 ± 0.07 F: 0.19 ± 0.06	M: 0.24 ± 0.10 F: 0.25 ± 0.10	M: 0.20 ± 0.08 F: 0.23 ± 0.07	0.376	0.249	0.418	0.016^8^	0.828
	WP	M: 0.17 ± 0.06 F: 0.16 ± 0.03	M: 0.17 ± 0.05 F: 0.17 ± 0.02	M: 0.15 ± 0.06 F: 0.17 ± 0.04	M: 0.18 ± 0.09 F: 0.20 ± 0.06					
SOST (pg/mL)	GY^6^	M: 490 ± 119	M: 374 ± 120	M: 426 ± 101	M: 437 ± 143	0.0005^8^	0.019^8^	0.994	0.004^8^	0.109
		F: 350 ± 61	F: 333 ± 81	F: 356 ± 68	F: 397 ± 104					
	WP^7^	M: 428 ± 106	M: 450 ± 74	M: 426 ± 108	M: 494 ± 120					
		F: 313 ± 75	F: 330 ± 62	F: 310 ± 77	F: 367 ± 47					
RANKL	GY	M: 100 ± 25 F: 85 ± 27	M: 92 ± 24 F: 88 ± 28	M: 94 ± 18 F: 104 ± 40	M: 98 ± 29 F: 103 ± 35	0.262	0.845	0.157	0.201	0.533
	WP	M: 128 ± 25 F: 79 ± 29	M: 112 ± 45 F: 71 ± 19	M: 114 ± 36 F: 67 ± 15	M: 120 ± 48 F: 80 ± 16					

Data are displayed as mean ± SD. GY group (*n* = 24, 13 males); WP group (*n* = 23, 13 males).

Abbreviations: CTX-I, C-terminal telopeptide of type I collagen; F, female; GY, Greek yogurt; IGF-1, insulin-like growth factor 1; M, male; OC, osteocalcin; OPG, osteoprotegerin; P1NP, procollagen type I N-terminal propeptide; RANKL, receptor activator of nuclear factor κΒ ligand; SOST, sclerostin; WP, whey protein.

1 Significantly higher in males across time (main sex effect).

2 Significantly lower than week 0 in GY males (time × group × sex interaction).

3 significantly higher than week 8 in GY males (time × group × sex interaction).

4 Significantly lower (GY) or higher (WP) in females (time × group × sex interaction).

5 Significantly higher at week 16 than week 8 within this group (time × group interaction).

6 Significantly lower at week 8 than week 0, returning to baseline levels from week 8 to week 24 within this group (time × group interaction).

7 Significantly higher at week 24 compared with all other times within this group (time × group × sex interaction).

8 Significant *P* values (*P*< 0.05).

**FIGURE 2:**
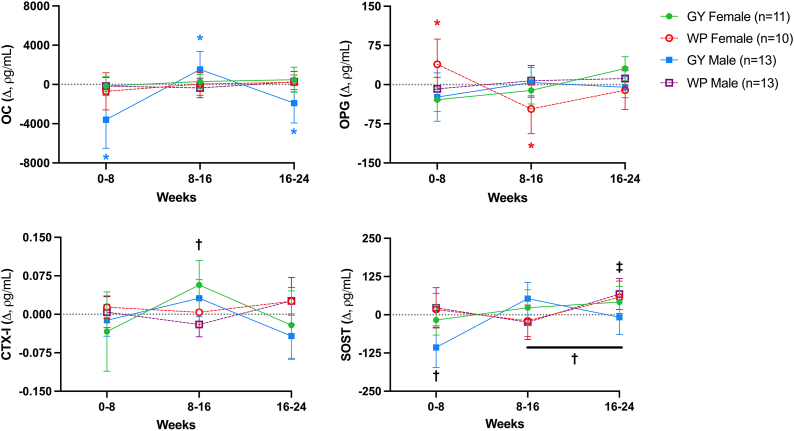
Differences in absolute concentrations of markers and osteokines of bone turnover that showed a significant time × group × sex interaction, namely Osteocalcin (OC, top-left), osteoprotegerin (OPG, top-right), or a significant time × group interaction, namely C-terminal telopeptide of type I collagen (CTX-I, bottom left), and sclerostin (SOST, bottom-right). Data are presented as mean ± SD of percent change (Δ) from week 0 to 8 (i.e., control period), week 8 to week 16 (i.e., post control to mid-intervention), and week 16 to week 24 (i.e., mid- to post intervention) for females and males within the Greek yogurt (GY) and whey protein (WP) groups. ∗denotes significant change within the sex-specific group of the same color (time × group × sex interaction). †denotes significant change within the GP group with sexes combined (time × group interaction). ‡denotes significant change within the WP group with sexes combined (time × group interaction).

After controlling for baseline concentrations and training volume, OC demonstrated a significant time × group × sex interaction (*F* = 4.52, *P* = 0.005) (Table 3). Post hoc analyses indicated that this interaction was driven exclusively by males in the GY group, who exhibited significant time-dependent fluctuations in OC (Table 3). Specifically in GY males (Figure 2), OC declined during the control period (weeks 0–8, *P* < 0.001), increased during early intervention (weeks 8‒16, *P* = 0.030), decreased during the second half of the intervention (weeks 16‒24, *P* = 0.009) to levels lower than its baseline levels at week 16 (*P* = 0.005) and week 24 (*P* < 0.001). In contrast, OC concentrations remained stable across time in GY females and in both male and female WP participants, with no significant within-group changes observed and no significant between-group differences at any time point (Figure 2). OPG response was also characterized by a significant time × group × sex interaction (*F* = 4.00, *P* = 0.009) driven by group-dependent fluctuations within the female participants (Table 3). In GY females, OPG concentrations showed minimal fluctuation over time, with only the early intervention levels at week 16 being lower than baseline (week 0; *P* = 0.046). In contrast, as seen in Figure 2, OPG concentrations in WP females exhibited more pronounced variation, increasing from week 0 to week 8 (*P* = 0.025) before returning to near-baseline values later in the intervention period (weeks 8‒16, *P* = 0.008; weeks 8‒24, *P* = 0.002). No significant changes in OPG were observed in males, irrespective of supplementation group (Figure 2).

CTX-I demonstrated a significant time × group interaction (*F* = 3.55, *P* = 0.016), indicating distinct temporal responses between the GY and WP groups (Table 3). In the GY group (males/females combined), CTX-I increased from week 8 to week 16 (*P* = 0.018), representing a transient rise during the early intervention phase (Figure 2), followed by a return toward baseline levels by week 24 (*P* = 0.072). In contrast, CTX-I concentrations in the WP group remained stable across all time points, with no significant temporal variation observed (Figure 2). SOST also showed a significant time × group interaction (*F* = 5.02, *P* = 0.004), indicating differential temporal responses between the supplementation groups (Table 3). As presented in Figure 2, in the GY group (males/females combined), SOST concentrations declined during the control period (weeks 0‒8, *P* = 0.002), then after a nonsignificant increase from week 8 to week 16 (*P* = 0.309), it returned to baseline levels during the intervention (weeks 8‒24, *P* = 0.002). In the WP group, SOST was stable during the control period and until week 16, but then increased thereafter (weeks 16‒24, *P* = 0.002) to levels higher than baseline by week 24 (weeks 0‒24, *P* = 0.002) (Figure 2). Finally, RANKL concentrations remained stable across time in all groups and sexes, with no meaningful temporal changes detected (Table 3). However, there was a significant group × sex interaction (*F* = 4.20, *P* = 0.046) with post hoc analyses indicating that females in the GY group exhibited consistently higher RANKL concentrations compared with WP females, with no between-group differences in males (Table 3).

### Markers of inflammation

**FIGURE 3:**
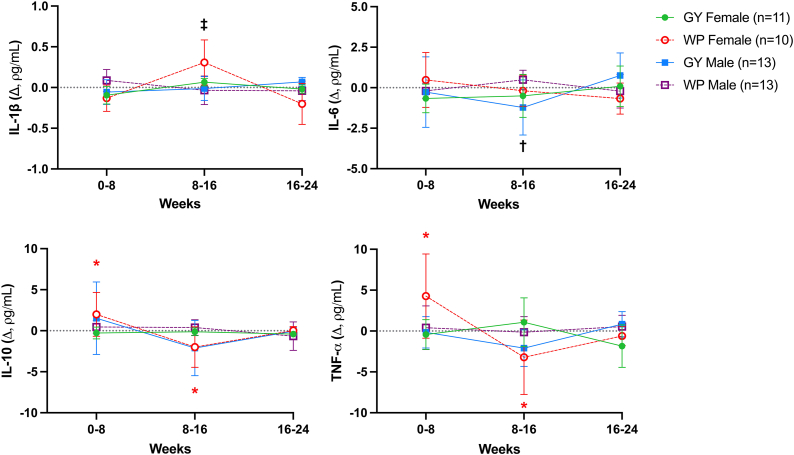
Differences in absolute concentrations of pro- and anti-inflammatory cytokines that showed a significant time × group interaction, namely IL-1β (top-left), IL-6 (top-right), or a significant time × group × sex interaction, namely IL-10 (bottom left), and TNF-α (bottom-right). Data are presented as mean ± SD of percent change (Δ) from week 0 to week 8 (i.e., control period), week 8 to week 16 (i.e., post control to mid-intervention), and week 16 to week 24 (i.e., mid- to post intervention) for females and males within the Greek yogurt (GY) and whey protein (WP) groups. ∗Denotes significant change within the group of the same color (time × group × sex interaction). †Denotes significant change within the GP group with sexes combined (time × group interaction). †Denotes significant change within the WP group with sexes combined (time × group interaction).

Absolute concentrations of IL-1β, IL-6, IL-10, and TNF-α across the season are presented in Table 4. After controlling for baseline concentrations and training volume, a significant time × group interaction was observed in IL-1β (*F* = 3.64, *P* = 0.023) (Table 4). This interaction reflects elevated concentrations in early intervention (week 16) compared with week 0 (*P* = 0.005), week 8 (*P* = 0.006), and week 24 (*P* = 0.041) in WP (males/females combined), which was not significant in GY (Figure 3). A significant time × group interaction was also observed (*F* = 2.84, *P* = 0.040) in IL-6 (Table 4), which was lower at week 16 compared with week 0 in GY (*P* = 0.006), whereas it remained stable in WP (Figure 3).

**TABLE 4:** Concentrations of pro- and anti-inflammatory cytokines at weeks 0, 8, 16, and 24 for male and female athletes in the Greek yogurt and whey protein groups

		Week 0	Week 8	Week 16	Week 24	Time	Group	Sex	Time × group	Time × group × sex
IL-1β (pg/mL)	GY	M: 0.31 ± 0.20 F: 0.23 ± 0.14	M: 0.31 ± 0.25 F: 0.14 ± 0.07	M: 0.22 ± 0.16 F: 0.21 ± 0.07	M: 0.36 ± 0.24 F: 0.19 ± 0.11	0.083	0.003^4^	0.602	0.023^4^	0.415
	WP^1^	M: 0.29 ± 0.09 F: 0.37 ± 0.15	M: 0.38 ± 0.21 F: 0.29 ± 0.22	M: 0.45 ± 0.38 F: 0.60 ± 0.35	M: 0.33 ± 0.18 F: 0.40 ± 0.12					
IL-6 (pg/mL)	GY^2^	M: 2.99 ± 2.81 F: 2.77 ± 2.56	M: 2.73 ± 2.50 F: 2.11 ± 1.65	M: 1.50 ± 0.94 F: 1.60 ± 1.47	M: 2.25 ± 2.10 F: 1.69 ± 0.95	0.195	0.719	0.999	0.040^4^	0.626
	WP	M: 1.61 ± 0.58 F: 1.93 ± 1.57	M: 1.42 ± 0.71 F: 2.41 ± 1.32	M: 1.91 ± 1.17 F: 2.22 ± 1.46	M: 1.70 ± 1.22 F: 1.55 ± 0.49					
IL-10 (pg/mL)	GY	M: 2.84 ± 0.91 F: 3.19 ± 0.81	M: 2.93 ± 0.84 F: 3.13 ± 0.74	M: 2.33 ± 0.60 F: 3.00 ± 0.67	M: 2.40 ± 0.55 F: 2.59 ± 0.62	0.004^4^	0.032^4^	0.231	0.016^4^	0.046^4^
	WP	M: 2.81 ± 0.55 F: 2.26 ± 0.38	M: 2.94 ± 0.87 F: 3.59 ± 1.03^3^	M: 2.94 ± 0.87 F: 2.66 ± 0.82	M: 3.01 ± 0.72 F: 2.69 ± 0.62					
TNF-α (pg/mL)	GY	M: 15.6 ± 3.2 F: 12.6 ± 2.9	M: 15.5 ± 3.7 F: 12.2 ± 1.6	M: 13.4 ± 2.9 F: 13.2 ± 4.4	M: 14.2 ± 2.5 F: 11.4 ± 1.9	0.085	0.014^4^	0.908	0.095	0.039^4^
	WP	M: 14.4 ± 3.0 F: 12.9 ± 3.5	M: 14.8 ± 4.7 F: 17.2 ± 5.4^3^	M: 14.6 ± 3.5 F: 14.1 ± 3.0	M: 15.2 ± 3.9 F: 13.5 ± 2.8					

Data are displayed as mean ± SD. GY group (*n* = 24, 13 males); WP group (*n* = 23, 13 males).

Abbreviations: F, female; GY, Greek yogurt; M, male; WP, whey protein.

1 Significantly higher within this group (time × group interaction).

2 Significantly lower within this group (time × group interaction).

3 Significantly higher at week 8 compared with all other times in WP females (time × group × sex interaction).

4 Significant *P* values (*P*< 0.05).

As shown in Table 4, IL-10 demonstrated a significant time × group × sex interaction (*F* = 2.75, *P* = 0.046). This interaction was driven primarily by females in the WP group, who exhibited a marked increase in IL-10 during the control period, i.e., before supplementation (weeks 0‒8, *P* < 0.001), followed by a return toward baseline from week 8 to week 16 (*P* = 0.002) weeks 8‒24 (*P* = 0.010) (Figure 3). In contrast, IL-10 concentrations remained relatively stable across time in males and in GY females (Figure 3). Likewise, TNF-α showed a significant time × group × sex interaction (*F* = 2.86, *P* = 0.039), reflecting sex-dependent temporal responses that differed by supplementation group (Table 4). Females in WP presented a significant increase in TNF-α concentrations at the end of the control period (weeks 0‒8, *P* < 0.001), which returned to baseline levels in the following 16 wk (*P* < 0.001) of the intervention (Figure 3).

## Discussion

This study compared the effects of GY with WP supplementation on markers of bone turnover and inflammation in adolescent athletes across a competitive season. Although the interventions were matched for protein, they differed in nutrient composition and food matrix, allowing comparison of a wholefood dairy source and an isolated protein supplement within the context of ongoing growth and seasonal training. Contrary to our hypothesis, GY supplementation did not consistently produce greater changes in markers of bone turnover or inflammation compared with WP. Rather, both protein sources were associated with modest, somewhat different (CTX-I and SOST), and often sex-specific (OC and OPG) effects. Inflammatory cytokines demonstrated dynamic variation across the competitive season, potentially driven by fluctuations in training intensity, with protein sources exerting limited, often conflicting, effects. These findings extend prior work from our group in adolescent female athletes, where we demonstrated that short-term GY intake during a week-long intensified training program primarily influenced indices of recovery and inflammation [17]. Herein, we demonstrate that longer-term supplementation across a competitive season is associated with nuanced, sex-dependent skeletal and inflammatory trajectories rather than uniform effects across outcomes.

### Nutritional and body composition changes

The GY and WP supplements were purposely matched for protein content, but neither successfully increased daily protein intake. This does not align with the compliance scores between groups assessed at week 16 and week 24. The lack of a statistically significant increase in protein intake following supplementation may be explained by relatively high protein intakes at baseline, and the considerable interindividual variability in self-reported dietary data, a trend that is similarly reflected in the observed fluctuations of total energy and carbohydrate intake. In fact, overall energy intake was either maintained or decreased throughout the season regardless of the added calories of the supplements. Thus, it is possible that the athletes inadvertently adjusted their overall food consumption to offset the additional calories from supplementation, potentially due to concerns about weight gain.

Irrespective of the overall stable dietary intake, body mass increased over time with a significant time × sex interaction reflecting sex-specific trajectories of mass accretion during adolescence, consistent with established patterns of pubertal growth and lean mass development in youth athletes [[23], [24], [25]]. In addition, no supplement-related interactions were observed. This finding is consistent with evidence that changes in body mass during adolescence are largely driven by biological maturation and sport-specific training exposure, which may diminish the likelihood of detecting effects from isolated dietary interventions [23,26]. Finally, females had higher %BF than males, a pattern well documented during adolescence and competitive sport participation [1,23,24,27].

### Markers of bone formation

OC demonstrated a significant time × group × sex interaction that was driven exclusively by males in the GY group, who exhibited clear temporal changes (decreases and increases) in OC across the season. This finding suggests that bone turnover dynamics in adolescent males may be particularly sensitive to the interaction between seasonal training stress and dietary protein consumed within a wholefood dairy matrix. Interestingly, a decrease in OC was also found in adolescent females with overweight and obesity who received additional servings of dairy combined with exercise over a 12-wk period [28]. Adolescence is characterized by elevated baseline bone remodeling rates and heightened responsiveness to mechanical loading, and prior work in adolescent and high-impact sport athletes has shown that OC is especially sensitive to training volume and competitive season demands, with both increases and decreases reported depending on training load, maturation, and timing within the competitive season [1,5,23,24,27,29]. The present findings extend this literature by indicating that the training season OC response may be more pronounced when protein is delivered within a fermented dairy matrix such as GY. In contrast, adult trials examining dairy or protein supplementation during exercise training have generally reported weak or null effects on OC [20,[30], [31], [32]], underscoring the heightened physiological plasticity of the adolescent skeleton relative to mature bone.

Male adolescent athletes exhibited higher concentrations of P1NP than females across all time points, but no significant effects of time, supplementation group, or higher-order interactions. These findings are consistent with established sex-specific differences in collagen synthesis and skeletal growth during adolescence [23,27,33]. Substantial interindividual variability in circulating P1NP has also been reported in physically active populations, with heterogeneous or no response observed even under controlled exercise conditions [1,28,34]. Indeed, P1NP has not responded to previous dairy-based interventions combined with exercise training in either young adult athletes [20] or adolescent girls with overweight and obesity [28]. Together, these findings suggest that circulating P1NP, specifically in adolescents, may be relatively insensitive to seasonal supplementation effects within an already active cohort, potentially due to ceiling effect [35].

IGF-1 concentrations remained stable across time, group, and sex, with no significant effects or interactions detected. However, unlike all other biomarkers, training volume emerged as a significant covariate, indicating that IGF-1 tracked more closely with mechanical and metabolic loading than with protein source. This finding aligns with literature indicating that IGF-1 is strongly influenced by other factors, including training load and energy availability, which were not assessed in our study, rather than short-term nutritional interventions [21,25,36].

OPG showed a significant time × group × sex interaction driven by changes in the female adolescent athletes. Females in both the GY and WP groups exhibited temporal changes in OPG across the competitive season, whereas males showed no significant variation at any time point. Specifically, female athletes in the GY group had lower OPG during early intervention (week 16) compared with their baseline, and those in the WP group had higher OPG at the end of the control period that dropped back to baseline levels thereafter. This highly variable sex-specific response is puzzling but may reflect that osteoclast regulatory pathways in adolescent females are sensitive to fluctuations in training load and/or hormonal changes [1,21,23]. Prior work suggests that OPG regulation is influenced by both mechanical loading and endocrine status, with training-induced changes reported more consistently in females than males [37,38]. Although adult supplementation trials generally report small or null effects of dairy or protein intake on OPG [20,30], the present findings indicate that OPG regulation in adolescent females may be more dynamic and responsive to training stressors and dietary intake.

### Markers of bone resorption

CTX-I exhibited a significant time × group interaction (*F* = 3.55, *P* = 0.016), with temporal changes occurring only in the GY group. Specifically, CTX-I increased with GY supplementation from week 8 to week 16 before declining toward week 24, indicating a transient rise in bone resorption during the early intervention phase followed by stabilization. This finding is consistent with our results in adult athletes, in whom CTX-I changes were also observed exclusively in the GY group [20]. However, in the adult cohort, the GY group showed an initial rise in CTX-I during the control period (weeks 0‒8), followed by a return to baseline by week 24 [20], probably reflecting adaptive remodeling responses to intensified training and short-term uncoupling of bone turnover [24,39,40]. The absence of comparable CTX-I changes in the WP group suggests that wholefood dairy intake may interact with training demands to influence resorptive dynamics.

In contrast to our adult athlete study, which reported no intervention effects on SOST with either GY or WP [20], in the present adolescent cohort, we found significant time-dependent fluctuations in SOST, with trajectories differing by dietary group. In the GY group, SOST concentrations declined during the control period, but after a nonsignificant increase during the first half of the intervention, returned to baseline levels by week 24. In contrast, the WP group exhibited a delayed SOST response, with stable concentrations during the control period and through mid-intervention, followed by an increase to above baseline levels at week 24. Given that SOST is highly responsive to mechanical loading and training status [38,[40], [41], [42]], these findings likely reflect training intensity-driven adaptations (not accounted for in this study) rather than isolated dietary effects. One previous 12-wk intervention combining dairy and exercise in adolescent females did not demonstrate an effect on SOST; however, this study was conducted in a nonathletic population with overweight and obesity, limiting its comparability [28]. To our knowledge, no studies have examined nutritional influences on SOST in adolescent athletes, highlighting the need for further work to clarify how nutrition and training load fluctuations interact with Wnt signaling during skeletal maturation.

The absence of temporal effects in RANKL aligns with prior trials reporting limited modulation of circulating RANKL [30,43,44]. However, in our adult athletes, RANKL increased from pre- to post control (weeks 0–8), indicating an initial upregulation of osteoclast signaling with the onset of training [20]. Thus, the absence of such a response in adolescents underscores population-specific RANKL responses or differences in training load periodization in youth sports. Moreover, the significant group × sex interaction suggests that adolescent females may exhibit greater sensitivity to dietary context, specifically to wholefoods such as GY, within osteoclast signaling pathways [38].

### Markers of inflammation

Circulating IL-1β displayed a significant time × group interaction across the competitive season, driven by a mid-season increase in the WP group and relative stability in the GY group. In WP, IL-1β concentrations were elevated at week 16 in both males and females before returning toward baseline by week 24, whereas no significant changes were observed in GY. It is previously suggested that IL-1β functions as a stress-responsive cytokine that reflects short-term immune activation during periods of intensified training rather than sustained changes in resting inflammatory status [45,46]. The absence of a comparable mid-season rise in the GY group suggests that fermented wholefood dairy intake may be associated with a more stable inflammatory profile during peak training demands, aligning with evidence that dairy consumption does not exacerbate systemic inflammation and may attenuate exercise-induced IL-1β responses [12,18]. Furthermore, circulating IL-6 concentrations decreased mid-intervention (week 16) in the GY group, and remained lower at the end of the supplementation compared with the control period, whereas concentrations remained stable across time in the WP group (time × group). This finding suggests a pro-inflammatory suppression effect of GY that was not observed in WP. However, in adult athletes, systemic IL-6 responses primarily reflect acute exercise exposure and metabolic stress, rather than chronic nutritional manipulation or longer-term training status [20,46,47]. In addition, although acute elevations in IL-6 following exercise are well documented, they typically resolve rapidly during recovery, limiting the likelihood of detecting sustained changes in resting concentrations across a season [46,48]. Nevertheless, the decrease in the resting concentrations of IL-6 with GY supplementation in our athletes is an interesting finding that aligns with Fraschetti et al. [48] 2025, who also reported a reduction in IL-6 following GY supplementation combined with resistance exercise in young adult, nonathletic males.

In contrast, TNF-α, the most commonly measured pro-inflammatory cytokine, exhibited a significant time × group × sex interaction, revealing complex, sex-dependent inflammatory trajectories across the competitive season. Specifically, TNF-α increased during the control period but decreased with WP supplementation only in females. This finding aligns with our previous observation in young adult athletes of both sexes [20], but contrasts with adult intervention studies in nonathletes, which report minimal or null TNF-α responses to exercise training or protein and dairy supplementation [[48], [49], [50], [51]]. The greater variability observed in female adolescent athletes, although only seen in the WP group, may reflect heightened immune plasticity during growth and maturation, when inflammatory regulation is more tightly coupled to training stress, recovery capacity, and hormonal variability across the menstrual phases [17,46,52]. Sex-specific immune signaling differences during adolescence may further contribute to divergent TNF-α responses, particularly under sustained competitive and physiological stress.

Finally, the anti-inflammatory cytokine IL-10 had a response characterized by pronounced time-, group-, and sex-dependent effects across the competitive season. In fact, only the females in the WP group exhibited an increase in IL-10 during the control period (week 0 to week 8), which could be a response to the simultaneous increase in TNF-α in this same group, followed by a return toward baseline at weeks 16 and 24. Similarly to stable IL-10 concentrations found in our GY group and the WP males, previous adult studies, including athletes, report largely null effects of dairy intake or dietary manipulation on IL-10 concentrations [20,48,49,53]. Thus, the magnitude and variability of both IL-10 and likely also TNF-α responses observed in our WP female cohort may be attributed to training-related differences in this group, supporting the concept that during adolescence, anti-inflammatory pathways may be more responsive to training load, recovery demands, and nutritional context [52]. One previous study incorporating GY in adolescent athletes during a week of intensified training reported alterations in indices of recovery, alongside limited or short-lived changes in resting concentrations of inflammatory cytokines, specifically IL-10 [17].

### Strengths and limitations

The strengths of the present study include its longitudinal design spanning 1 competitive training season, the incorporation of a within-subject control period, and an ecologically valid approach that allowed athletes to maintain their habitual training and dietary practices. A notable contribution is the direct comparison of a wholefood fermented dairy product with an isolated protein supplement, matched for protein content.

The study also had several limitations, with the most important being the absence of a nonsupplement control group, restricting the ability to fully separate the effects of protein supplementation from training-induced adaptations. This warrants cautious interpretation of the findings. Future research should incorporate a non-nutritive placebo-controlled design to clarify these effects. Additional limitations include the incorporation of youth athletes from multiple sports, along with the lack of information on training intensity (which likely varied by sport), which may influence and potentially explain the observed bone and immune responses, or the absence thereof. To maintain ecological validity, diets were also not nutritionally standardized between groups, which may have influenced outcomes. Likewise, we did not account for menstrual cycle phase in females, which could explain variation in values and limit the ability to detect significant differences between groups, especially in CTX-I since it has been found to be lower during the luteal phase [54]. However, other studies found no fluctuations during the menstrual cycle in resting concentrations of CTX-I, P1NP, or SOST [55,56]. Finally, the inclusion of mixed-sex groups may have introduced baseline variability and reduced statistical power to detect interaction effects. Although this approach enhances generalizability and enables exploration of sex differences, it also adds complexity that future studies should address.

In conclusion, overall, these findings indicate that adolescent athletes exhibit dynamic, often sex-specific bone turnover across a competitive training season, with GY and WP exerting modest, but somewhat different (CTX-I and SOST), and often sex-specific (OC and OPG) effects. Likewise, pro-inflammatory cytokines (IL-1β, IL-6, and TNF-α) and anti-inflammatory IL-10, displayed complex sex- and group-dependent trajectories mainly suggesting that protein supplementation, independent of source (wholefood or isolate), may interact with training-induced inflammation in adolescent athletes. Collectively, these results suggest that adolescence is a dynamic period during which growth- and maturation-related factors (which differ between males and females), combined with fluctuations in training intensity, may outweigh the potential effects of nutritional supplementation in adolescent athletes. Future studies incorporating maturity status, assessment of acute biomarker responses to exercise, and longer intervention durations are warranted to further clarify the role of wholefood dairy in supporting bone and immune health during adolescents’ athletic development.

## Author contributions

The authors’ responsibilities were as follows–MB, BDR, ARJ, BF, PK: designed research; MB, PHN, EM, PK: conducted research; MB, PHN: analyzed data; MB, PHN, EM: wrote the paper; PK: had primary responsibility for final content; and all authors: read and approved the final manuscript.

## Declaration of generative AI and AI-assisted technologies in the writing process

The authors declare that no generative AI or AI-assisted technologies were used in the writing of this manuscript.

## Data availability

Data described in the manuscript will be made available upon reasonable request to the corresponding author.

## Funding

The study received grant funding from the Dairy Farmers of Canada to PK. The funders had no role in the design, execution, interpretation, or writing of the study. MB and MIR were supported by a Mitacs fellowship.

## Conflict of interest

ARJ reports a relationship with Dairy Farmers of Canada that includes consulting/advisory fees. All other authors report no conflicts of interest.
